# Evaluation of exacerbations and blood eosinophils in UK and US COPD populations

**DOI:** 10.1186/s12931-019-1130-y

**Published:** 2019-08-07

**Authors:** Claus F. Vogelmeier, Konstantinos Kostikas, Juanzhi Fang, Hengfeng Tian, Bethan Jones, Christopher Ll Morgan, Robert Fogel, Florian S. Gutzwiller, Hui Cao

**Affiliations:** 1 Department of Medicine, Pulmonary and Critical Care Medicine, University Medical Centre Giessen and Marburg, Philipps-University Marburg, Member of the German Center for Lung Research (DZL), 35043 Marburg, Germany; 2 Respiratory Medicine Department, University of Ioannina School of Medicine, Ioannina, Greece; 3 Novartis Pharmaceuticals Corporation, East Hanover, NJ USA; 4 KMK Consulting Inc, Morristown, NJ USA; 5 Pharmatelligence, Cardiff, UK; 6 Novartis Pharma AG, Basel, Switzerland

**Keywords:** COPD, Triple therapy, Eosinophil, Exacerbation

## Abstract

**Background:**

Blood eosinophil counts and history of exacerbations have been proposed as predictors of patients with chronic obstructive pulmonary disease (COPD) who may benefit from triple therapy (inhaled corticosteroid, long-acting β_2_-agonist and long-acting muscarinic antagonist).

**Methods:**

In a retrospective cohort analysis we examined the profiles of COPD patients from the UK Clinical Practice Research Datalink (CPRD) and US Optum Clinformatics™ Data Mart (Optum) databases with reference to exacerbation frequency and blood eosinophil distribution.

**Results:**

Of the 31,437 (CPRD) and 383,825 (Optum) patients with COPD, 15,364 (CPRD) and 139,465 (Optum) met the eligibility criteria and were included. Among patients with ≥2 exacerbations and available eosinophil counts in the baseline period (CPRD, *n* = 3089 and Optum, *n* = 13414), 17.0 and 13.3% respectively had eosinophil counts ≥400 cells/μL. Patients with ≥2 exacerbations or eosinophil count ≥400 cells/μL during first year, exacerbated at least once (CPRD, 82.8% vs Optum, 80.6%) or continued to have eosinophil count ≥300 cells/μL (76.8% vs 76.5%), respectively in the follow-up year. In both years, a higher variability in the number of exacerbations and eosinophil count was observed in patients with one exacerbation and eosinophil counts between 300 and 400 cells/μL; patients with eosinophil count < 150 cells/μL had the lowest variability. Approximately 10% patients had both ≥2 exacerbations and eosinophil count ≥300 cells/μL across the databases.

**Conclusion:**

A high variability in blood eosinophil counts over two consecutive years was observed in UK and US patients with COPD and should be considered while making treatment decisions. A small proportion of COPD patients had frequent exacerbations and eosinophil count ≥300 cells/μL.

**Electronic supplementary material:**

The online version of this article (10.1186/s12931-019-1130-y) contains supplementary material, which is available to authorized users.

## Background

Blood eosinophil counts and history of exacerbations have been proposed as predictors of patients with chronic obstructive pulmonary disease (COPD) who may benefit from triple therapy (inhaled corticosteroids [ICS] plus long-acting β_2_-agonist [LABA] plus long-acting muscarinic antagonist [LAMA]). Recently, studies have showed that triple therapy provides better exacerbation prevention in frequently exacerbating patients (≥2 moderate/severe exacerbations or one hospitalisation) and in those with higher blood eosinophil count (e.g. ≥300 cells/μL) vs dual bronchodilation with LABA plus LAMA [1, 2]. In these studies patients with previous asthma were allowed [1] or the proportion of patients with frequent exacerbations and high eosinophil count constituted about 5 to 10% of the total study population [1, 2]. Retrospective studies in clinical practice have shown that a substantial proportion of patients receive triple therapy [3–5], suggesting a large number of patients with COPD receive ICS irrespective of their exacerbation status.

A post hoc analysis of the WISDOM study demonstrated that stopping ICS in patients on triple therapy may increase the rate of exacerbations in patients with high eosinophil count (≥300 cells/μL) and frequent exacerbations (≥2 exacerbations/year) [6]. Additionally, the SUNSET study demonstrated that nonfrequent exacerbators with low eosinophil count (≤300 cells/μL) on long-term triple therapy (tiotropium plus salmeterol/fluticasone) can be switched to indacaterol/glycopyrronium without increasing the exacerbation risk [7].

Accumulating evidence suggests that identifying the right COPD patients for long-term triple therapy would enable more personalised care. Based on the GOLD strategy document and recent studies, the patients who would benefit the most from ICS use in terms of exacerbation prevention would be those with elevated blood eosinophil levels, as well as those and with frequent exacerbations. In this analysis, we estimated the proportion of patients with frequent exacerbations and higher blood eosinophil count, and stability of these characteristics over 2 years in United Kingdom (UK) and United States (US) populations in order to identify patients who are most likely to benefit from ICS.

## Methods

We conducted a retrospective cohort analysis using two databases: Clinical Practice Research Datalink (CPRD) with linked Hospital Episode Statistics (HES) databases of primary and secondary care records from the UK [8], and the Optum Clinformatics™ Data Mart (Optum), a database of de-identified administrative claims from a commercially insured population in the US [9].

### Databases

CPRD, formerly known as the General Practice Research Datalink (GPRD), is a database of linked, anonymised, primary medical care records from patients treated at nearly 700 primary care practices in the UK since 1987. This population is considered representative of the UK primary care population in terms of age and sex, compared with the UK census of 2011 [10]. CPRD contains data from approximately 8% of the total UK population and it reflects the complete Electronic Medical Records (EMR) for all of the National Health Service (NHS) primary health care. Patient records at approximately 60% of the practices in CPRD are linked to HES, which provides data on all inpatient and outpatient contacts occurring within National Health Service hospitals in the UK [8].

OPTUM is a database of administrative health claims for members of United Healthcare, a large American for-profit managed care company. This database includes data of approximately 12 to 14 million annual covered lives. The OPTUM was statistically de-identified under the Expert Determination method, meeting the requirements of the Health Insurance Portability and Accountability Act (HIPAA). The claims data comprised both commercial and Medicare Advantage health plan data of the population that is geographically diverse spanning over all 50 states in the US [9].

### Study population

The study population comprised two COPD cohorts according to the index year each with 01 January as the index date: the 2014 cohort and the 2015 cohort. The baseline period was 2013 and 2014 for the 2014 and 2015 cohorts, respectively (Additional file 1: Figure S1).

Patients (men or women, aged ≥40 years at index) must have at least two outpatient diagnoses of COPD (at least 7 days apart) or one inpatient primary diagnosis of COPD in the baseline year, and at least 1 year of data pre- and post-index. Additionally, patients from CPRD were required to have HES linkage scheme allowing linkage to their secondary care records. Patients with a diagnosis of asthma in either the baseline or index year were excluded.

Besides, a subgroup of the 2014 cohort with patients having 2-year follow-up data was evaluated to assess the association of the outcomes during first follow-up and second follow-up. Data of the 2014 and 2015 cohorts were analysed separately and are reported.

### Study measures

The baseline demographics and clinical characteristics recorded and available for analysis at the index date or in the baseline period were collected. The characteristics included age and sex, socioeconomic status (CPRD only), smoking history (CPRD only), lung function (CPRD only) and the modified Medical Research Council (mMRC) scale (CPRD only). Additionally, patients from CPRD database were classified into four groups, i.e. GOLD A, B, C and D, based on a combination of severity, symptoms and previous exacerbations according to the GOLD 2017 document [11]. Commonly reported comorbidities in patients with COPD (hypertension, diabetes, acute myocardial infarction, heart failure, stroke, asthma, atrial fibrillation, depression, anxiety, osteoporosis, pneumonia, hyperlipidaemia and cancer in the pre-index date period) and the Charlson comorbidity index (CCI) were reported. Prescribed COPD medications in the baseline period were identified. The following treatments recommended as monotherapy or combination therapy by the GOLD 2017 guidelines were investigated: short-acting β_2_-agonist (SABA), short-acting muscarinic antagonist (SAMA), LABA, LAMA and ICS. Exacerbations in the baseline year were identified using a prespecified algorithm (Additional file 1: Table S1). Exacerbations in COPD patients were defined using a modified version of the algorithm developed by Mapel et al. [12] and Macaulay et al. [13] for the Optum database analysis and modified based on drug codes and diagnosis codes for the CPRD database analysis. Two exacerbation events occurring within 14 days were considered the same exacerbation.

Eosinophil count was identified within 6 months pre- or post-index date. The measure closest to the index was used when multiple eosinophil measures were found. The variable evaluated in the post-index period was number of exacerbations in the index year.

### Statistical analyses

Categorical variables were presented as count and percentage of patients in each category. Continuous variables were summarised by providing mean and standard deviation, median, quartiles Q1 and Q3 and the minimum and maximum values. For subgroup analysis, the number of exacerbations in the second year was cross-tabulated with the number of exacerbations during the first follow-up. In addition, for patients with ≥2 exacerbations during the first follow-up with eosinophil count ≥300 cells/μL or ≥ 400 cells/μL, the number of exacerbations (< 2 or ≥ 2) and/or eosinophil count (< 300 cells/μL or ≥ 300 cells/μL) at the second follow-up are presented. For variables such as FEV1% predicted, blood eosinophil count, etc., patients without missing data were reported.

## Results

### Cohort size

**Fig. 1:**
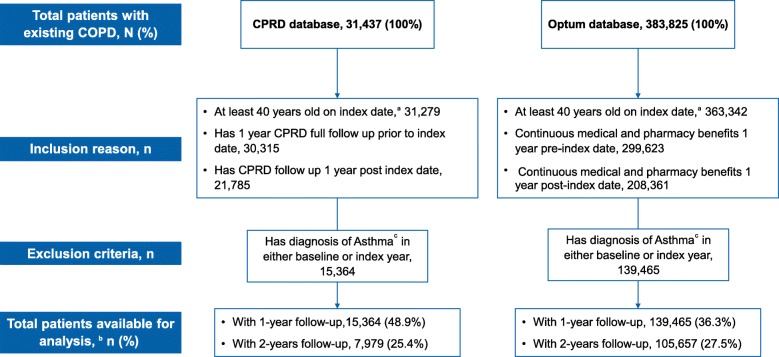
Patient flow. ^a^Jan 01, 2014 or Jan 01, 2015; ^b^Patients with at least one COPD diagnosis during the index year period and met the eligibility criteria; ^c^Asthma ICD-9-CM: 493.x, ICD-10-CM: J45x and J46x

The results from the 2014 cohort are presented here. A total of 15,364 patients from CPRD and 139,465 from Optum met inclusion and exclusion criteria. Two-year follow-up data were available for 7979 (52%, CPRD) and 105,657 (76%, Optum) patients (Fig. 1).

### Patient characteristics

Baseline demographics and clinical characteristics of patients from both the databases were comparable and are summarised in Table 1. In both CPRD and Optum databases, the majority of patients (> 75%) were aged ≥65 years, with 36.2 and 7.5% patients, respectively, on ICS + LABA + LAMA treatment. The most common comorbidity was hypertension, followed by hyperlipidaemia in both the databases. The patients without the follow-up year data were mainly due to lost to follow-up or were those who changed healthcare plan coverage. The demographic characteristics of patients with follow-up data were similar to those of the overall population (see Additional file 1: Table S2).

**Table 1:** Baseline demographics and clinical characteristics (2014 cohort)

Characteristic	CPRD database *N* = 15,364	Optum database *N* = 139,465
Age, years, mean (SD)	71.8 (10.4)	71.8 (10.6)
Gender		
Men	8,383 (54.6%)	71,417 (51.2%)
Women	6,981 (45.4%)	68,047 (48.8%)
Charlson Comorbidity Index^a^, median (IQR)	2.0 (1.0–4.0)	2.0 (0.0–3.0)
Baseline comorbidities common to COPD patients^b^		
Hypertension	9,133 (59.4%)	106,687 (76.5%)
Hyperlipidaemia	5,972 (38.9%)	91,779 (65.8%)
Depression	5,321 (34.6%)	26,030 (18.7%)
Anxiety	4,524 (29.4%)	23,138 (16.6%)
Cancer	3,915 (25.5%)	32,729 (23.5%)
Diabetes	2,755 (17.9%)	46,588 (33.4%)
Baseline medications^c^		
ICS	1,078 (7.0%)	5,900 (4.2%)
LABA	513 (3.3%)	1,556 (1.1%)
LAMA	3,658 (23.8%)	24,850 (17.8%)
LAMA + LABA	423 (2.8%)	615 (0.4%)
ICS + LABA	4,938 (32.1%)	29,763 (21.3%)
ICS + LAMA + LABA	5,563 (36.2%)	10,490 (7.5%)
Smoking status		
Patients with smoking data reported, n	15,356	–
Current smokers	4,996 (32.5%)	–
Ex-smokers	9,499 (61.8%)	–
Non-smokers	861 (5.6%)	–
GOLD classification^d^		
Patients with GOLD assessment, n	6,029	–
Group A	449 (7.4%)	–
Group B	1,843 (30.6%)	–
Group C	309 (5.1%)	–
Group D	3,428 (56.9%)	–
mMRC dyspnoea scale		
Patients with mMRC data, n	10,899	–
Grade 0	1,341 (12.3%)	–
Grade 1	3,941 (36.2%)	–
Grade 2	3,157 (29.0%)	–
Grade 3	2,033 (18.7%)	–
Grade 4	427 (3.9%)	–
Patients with FEV_1_ % predicted data	7,331 (47.7%)	–
FEV_1_, % predicted, mean (SD)	61.6 (21.6)	–
Patients with FEV_1_/FVC data	6,769 (44.1%)	–
FEV_1_/FVC, %, mean (SD)	60.2 (16.0)	–

Data are presented as n (%), unless specified otherwise

*COPD* chronic obstructive pulmonary disease, *CPRD* Clinical Practice Research Datalink, *FEV*_*1*_ forced expiratory volume in 1 second, *FVC* forced vital capacity, *GOLD* Global Initiative for Chronic Obstructive Lung Disease, *ICS* inhaled corticosteroid, *IQR* interquartile range, *LABA* long-acting β2-agonist, *LAMA* long-acting muscarinic antagonist, *mMRC* modified Medical Research Council, *SD* standard deviation

^a^Charlson Comorbidity Index comprises 19 comorbid disease categories, each assigned a score from 1 to 6, and is used to predict 10-year mortality in patients with comorbidities; the greater the score, the greater the risk of mortality

^b^Comorbidities present in ≥ 25% of the patients in either database are presented

^c^Patient was only counted if length of medication use was ≥ 30 days

^d^As per GOLD 2017 recommendations [10]

### COPD exacerbations

**Fig. 2:**
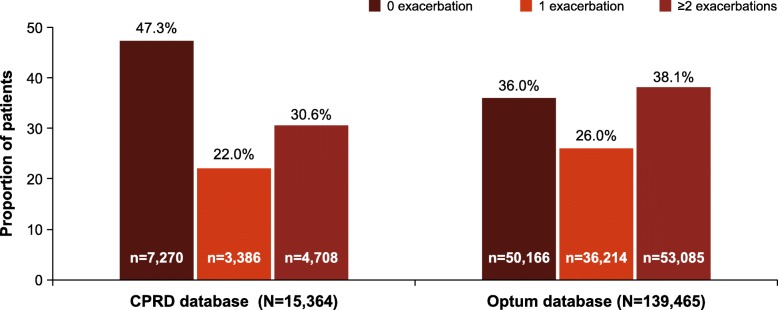
COPD patient population by exacerbation frequency in the index year (2014 cohort). CPRD, Clinical Practice Research Datalink; Optum, Optum Clinformatics™ Data Mart

Exacerbations for patients in both the databases are detailed in (Fig. 2).

**Fig. 3:**
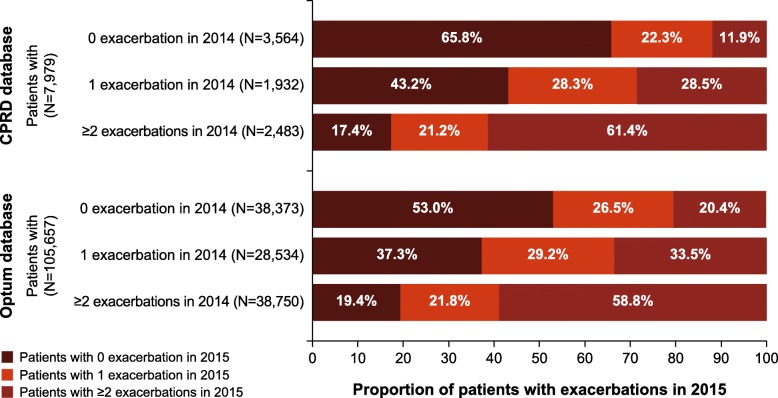
Distribution of exacerbations at the second follow-up according to exacerbation rates at the first follow-up (2014 cohort). CPRD, Clinical Practice Research Datalink; Optum, Optum Clinformatics™ Data Mart

Figure 3 illustrates the frequency of exacerbations in the second year as a function of exacerbations in the first follow-up, among patients with 2-year follow-up data. In both the databases, patients who had no exacerbations in the first year were less likely to experience two or more exacerbations at the second follow-up (12 to 20%). Patients who had experienced at least two exacerbations at the first follow-up were most likely to have exacerbations at the second follow-up (at least one exacerbation at the second follow-up for > 80% of patients); whereas this proportion varied for patients who had experienced one exacerbation at the first follow-up.

### Eosinophil count

**Fig. 4:**
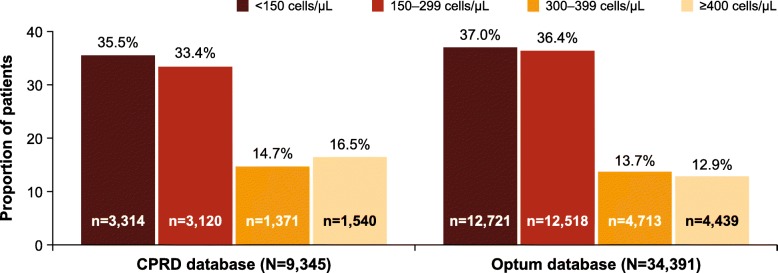
Study population by blood eosinophil count (2014 cohort). The closest eosinophil record within ± 180 days to the index date was used as the baseline value. CDM, Clinformatics™ Data Mart; CPRD, Clinical Practice Research Datalink

Figure 4 presents the proportion of patients with prior eosinophil count (for patients with 2-year follow-up data) according to eosinophil count in the second-year follow-up.

In the CPRD database, blood eosinophil counts were available for 9345 (60.8%) patients in 2014. Of these, 31.2% of patients had eosinophil count ≥300 cells/μL, while 16.5% had ≥400 cells/μL. In the Optum database, blood eosinophil counts were available for 34,391 (24.7%) patients in 2014. Of these, 26.6% of patients had eosinophil count ≥300 cells/μL, while 12.9% had ≥400 cells/μL.

**Fig. 5:**
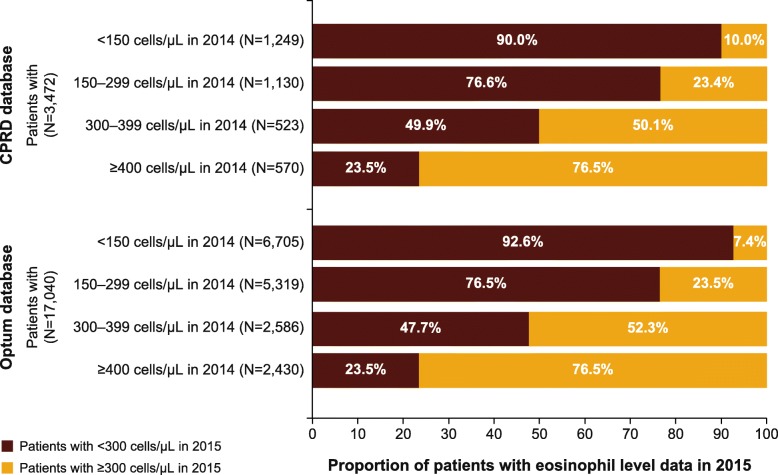
Proportion of patients according to their eosinophil count in the first and second years of follow-up (2014 cohort). CDM, Clinformatics™ Data Mart; CPRD, Clinical Practice Research Datalink

A total of 3472 and 17,040 patients in the CPRD and Optum databases, respectively, had 2-year follow-up data and eosinophils counts recorded in both the follow-up years (Fig. 5). The majority of patients (76.5%) with eosinophil count ≥400 cells/μL during the first follow-up continued to have elevated eosinophil count (≥300 cells/μL) at the second follow-up. In both databases, the great majority of patients who had eosinophil count < 300 cells/μL at the first follow-up continued to have low eosinophil count at the second follow-up (> 80% patients). For patients who had eosinophil count 300–399 cells/μL at the first follow-up the likelihood of having > = 300 cells/μL at the second follow up was approximately 50% (Fig. 5 and Additional file 1: Table S3).

### Exacerbations and blood eosinophil counts

The distribution of patients based on high exacerbations and eosinophil count indicated that although many COPD patients had either ≥2 exacerbations or eosinophil count of ≥300 or ≥ 400 cells/μL, only a small proportion of patients had both (approximately 10% had ≥2 exacerbations and eosinophil count of ≥300 cells/μL; approximately 5% had ≥2 exacerbations and eosinophil count of ≥400 cells/μL) (Table 2 and Additional file 1: Table S8).

**Table 2:** Distribution of patients based on high exacerbations and blood eosinophil counts (2014 cohort)

Population	CPRD database *N* = 15,364	Optum database *N* = 139,465
Patients with ≥ 2 exacerbations in index year, n	4,708	53,085
Patients with eosinophil count in baseline period, n	3,089 (65.6%)	13,414
< 150 cells/μL	1,093 (35.4%)	4,926 (36.7%)
150–299 cells/μL	1,024 (33.1%)	4,834 (36.0%)
300–399 cells/μL	448 (14.5%)	1,869 (13.9%)
≥ 400 cells/μL	524 (17.0%)	1,785 (13.3%)
Total patients with eosinophil count in baseline period, n	9,345	34,391
≥ 2 exacerbations and eosinophil count ≥300 cells/μL, n (%)	972 (10.4%)	3,654 (10.6%)
≥ 2 exacerbations and eosinophil count ≥400 cells/μL, n (%)	524 (5.6%)	1,785 (5.2%)

*CDM* Clinformatics™ Data Mart, *CPRD* Clinical Practice Research Datalink

The results from 2015 cohort were consistent with that of 2014 cohort and are presented in Additional file 1: Tables S4, S5, S6, S7, S8).

Further, to examine the relationship between the eosinophil count and exacerbations, we cross tabulated the frequency of eosinophil counts in 1st year and exacerbations in second year for the 2014 cohort. In both the databases, the finding suggests that there is no trend for association of eosinophil counts and exacerbations (Additional file 1: Table S9). However, since our study was not designed to assess the association of eosinophil count and exacerbation further studies are warranted to explore the correlation.

## Discussion

In this retrospective analysis from two large databases from the UK and the US, we evaluated the prevalence and variability of exacerbation frequency and blood eosinophil count that may inform treatment decisions in patients with COPD. Earlier, real-world studies have reported that in patients with COPD exacerbation frequency in a single year does predict long-term exacerbation rates in a graduated fashion and blood eosinophil concentrations can guide the selection of inhaler [14, 15]. To our knowledge, this is the first study to analyse characteristics and distribution of COPD patients with respect to exacerbation rates and blood eosinophil counts.

Overall, patient characteristics were consistent in both the databases. In both databases, approximately 80% of the patents with ≥2 exacerbations in the index year persistently exacerbated during the second follow-up. In addition, patients with either low (< 300 cells/μL) or high blood eosinophil count (≥400 cells/μL) in the index year continued to maintain the similar level of blood eosinophil count at the second follow-up. Approximately 10% of patients who were frequent exacerbators (≥2 exacerbations) and had high eosinophil count (≥400 cells/μL) in the index year, persistently have higher rates of exacerbations and high eosinophil count at the second follow-up. A higher variability in the frequency of exacerbations and eosinophil count in the second year was observed among patients who had either one exacerbation or whose eosinophil count ranged between 300 and 400 cells/μL.

A previous study in COPD population without excluding patients with concurrent asthma from the UK had reported 20% of patients with eosinophil counts, of which approximately 65% had eosinophil count ≥150 cells/μL [3]. In another study, 20% of patients had eosinophil count ≥300 cells/μL and 11% had ≥400 cells/μL [6]. Our study population had higher distribution of blood eosinophil count than that of previous studies.

Earlier studies have shown that a history of exacerbations is predictive of the future risk of exacerbations, [3–6, 16, 17] which was observed in our study as well. We found that frequent exacerbators (≥2 exacerbations) continued to be exacerbators (≥1 exacerbations) in the subsequent follow-up. Among patients with a history of one exacerbation, we observed variability in exacerbation rate in follow-up. These findings suggest a history of ≥2 exacerbation is a more reliable predictor of future exacerbations.

Despite the fact that studies have reported that eosinophilic airway inflammation in patients with COPD could be associated with exacerbations and responsivity to ICS therapy, consensus on specific and consistent cut-off to define the increased exacerbation risk is lacking. Multiple studies have reported that measure of blood eosinophil at a single time point may not be sufficient and might require additional follow-up examinations [18–20]. For instance, the ECLIPSE study reported that 51% of patients had a persistent eosinophil count of < 2% or ≥ 2% over a period of 3 years [20, 21]. Another study which measured eosinophil count at every 3 months over a period of 1 year, reported 65% of COPD patients with persistent blood eosinophil count of approximately 400 cells/μL [22, 23]. Additionally, another challenge is inconsistency in long-term stability of blood eosinophil counts. In a recent case-control study that reported trajectory of long-term stability of blood eosinophils using a cut-off of 340 cells/μL, the proportion of COPD patients with stable eosinophil counts ranged from 75% (at 1 year) to 35% (at 8 years) [18]. In our study, we observed persistency in high (400 cells/μL) or low (300 cells/μL) eosinophil counts within 2 years of follow-up; whereas patients who had eosinophil counts between 300 and 400 cells/μL showed a higher variability. This finding indicates a considerable proportion of patients will continue to have variable blood eosinophil counts over time.

Although debatable, some studies (COPDGene and ECLIPSE) [21, 24, 25] showed that patients with moderate-to-severe COPD and blood eosinophil counts ≥300 cells/μL are at an increased risk of future exacerbations. The Copenhagen general population study reported a 1.76-fold increased risk of severe exacerbations for COPD patients with blood eosinophil counts of > 340 cells/μL [26]. These findings suggest that patients with infrequent exacerbations and eosinophil counts above 300 cells/μL demonstrate fluctuations in manifestation of exacerbations and may require frequent eosinophil count monitoring to guide appropriate course correction in their medical management.

Recently the results of the IMPACT [1] and TRIBUTE [2] studies showed that triple therapy was more effective than dual bronchodilation in preventing exacerbations in patients who had frequent exacerbations (≥2) and higher (≥300 cells/μL) eosinophil counts. However, triple therapy could not reach significant difference compared with dual bronchodilator therapy in patients with low eosinophil and lower number of exacerbations, implying that patients with low eosinophil count may not require ICS-containing therapy. The results of the SUNSET study revealed that patients with COPD on long-term triple therapy without frequent exacerbations can be de-escalated to dual bronchodilator therapy without worsening exacerbations if they have had baseline eosinophil count < 300 cells/μl [7]. A post hoc analysis of the WISDOM trial reported that patients with both high eosinophil counts (≥300 cells/μL) and history of ≥2 exacerbations in the previous year had an increased rate of exacerbations after ICS withdrawal [6]. The recently updated GOLD strategy document recommends escalation or de-escalation of ICS therapy based on the exacerbation history, blood eosinophil count, history of pneumonias, appropriateness of initial ICS prescription and lack of response to ICS [27]. In our study also, we found that only 10% of patients had high eosinophil count and frequent exacerbations. Collectively, the evidence indicates that a minority of the patient population with a high exacerbation risk and higher eosinophil count may be the right population for ICS therapy. However, studies have reported the proportion of COPD patients receiving triple therapy in the range of 30 to 50%, indicating that triple therapy is currently overprescribed [3–5]. Our results are based on a real-life study population and support clinicians to understand the prevalence and stability of the frequent exacerbator phenotype, as well as the presence of high blood eosinophil and their combination, complementing the data from the major clinical trials.

Our study has both strengths and limitations. The major strength is that this is the first study to compare two large databases in US and UK primary care, and since patients with concurrent asthma were excluded, our study population represent the true COPD population. Moreover, to our knowledge, for the first time, a study has evaluated this population for two consecutive years. As with all database analyses, results of this study should also be interpreted with caution. We excluded patients who had an asthma-related clinical visit, emergency room visit or hospitalization in the last 2 years, but may have included patients who had a history of asthma but no asthma-related visits in the last 2 years. These patients were either in the asthma remission stage or have intermittent, mild asthma. The inclusion of these patients do not impact our analyses and conclusion. Another potential limitation is the fact that we have reviewed the baseline medications and we cannot account for potential changes or switches in treatment regimens during follow-up. Although the ICS treatment might affect eosinophil counts [28], the effect of ICS on blood eosinophils is minimal [29], so the eosinophil data are rather solid. Of course, changes in treatment regimens may have changed the exacerbation status of patients, but likely this was not different between patient groups. Moreover, patients from two commercial databases having specific data of interest (i.e. eosinophil counts) were included in this analysis; hence, patients with such specific data might not represent the general COPD population. Nevertheless, we believe that these results are a precursor for the further focused studies.

## Conclusions

Distribution of exacerbation frequency and blood eosinophil counts were very similar in both COPD populations (UK and US) examined. COPD patients with a history of ≥2 exacerbations per year were more likely to experience exacerbations in the following year. Eosinophil count ≥400 cells/μL in the previous year was a predictor of high eosinophil count (≥300 cells/μL) in the following year. Although many COPD patients had either ≥2 exacerbations or blood eosinophil counts ≥300 or ≥ 400 cells/μL, only a small percentage of patients had both. Considering the adverse events associated with ICS, a smaller target population for triple therapy and variability in blood eosinophil counts in patients with COPD, prospective studies with multiple assessment points are warranted to establish the role of triple therapy and implication of blood eosinophil counts in clinical practice. Our results support our understanding of the populations with treatable characteristics within the COPD patients and may serve as the basis for the development of treatment strategies.

## Additional file

Additional file 1: **Figure S1.** Study design. **Table S1.** Description of exacerbation algorithm. **Table S2.** Baseline demographics and clinical characteristics of 2 year follow-up subgroup of 2014 cohort. **Table S3.** Distribution of patients based on eosinophil counts (2014 cohort). **Table S4.** Patient flow (2015 cohort). **Table S5.** Baseline demographics and clinical characteristics. (2015 cohort). **Table S6.** COPD patient population by exacerbation frequency in the index year (2015 cohort). **Table S7.** Distribution of blood eosinophil count (2015 cohort). **Table S8.** Distribution of patients based on high exacerbations and eosinophil counts (2015 cohort). **Table S9.** Cross tabulation of frequency of eosinophil counts in 1st year and exacerbations in second year (2014 cohort). (DOCX 86 kb)

## Data Availability

The datasets used and/or analysed during the current study are available from the corresponding author on reasonable request.

## Abbreviations

CCI: Charlson comorbidity index
CDM: Clinformatics™ Data Mart
COPD: Chronic obstructive pulmonary disease
CPRD: Clinical Practice Research Datalink
EMR: Electronic Medical Records
FVC: Forced vital capacity
GOLD: Global Initiative for Chronic Obstructive Lung Disease
HES: Hospital Episode Statistics
HIPAA: Health Insurance Portability and Accountability Act
ICS: Inhaled corticosteroids
LABA: Long-acting β2-agonist
LAMA: Long-acting muscarinic antagonist
NHS: National Health Service
OPTUM: Optum Clinformatics™ Data Mart
SABA: Short-acting β2-agonist
SAMA: Short-acting muscarinic antagonist

## References

[1] Lipson DA, Barnhart F, Brealey N, Brooks J, Criner GJ, Day NC, Dransfield MT, Halpin DMG, Han MK, Jones CE (2018). Once-daily single-inhaler triple versus dual therapy in patients with COPD. N Engl J Med.

[2] Papi A, Vestbo J, Fabbri L, Corradi M, Prunier H, Cohuet G, Guasconi A, Montagna I, Vezzoli S, Petruzzelli S (2018). Extrafine inhaled triple therapy versus dual bronchodilator therapy in chronic obstructive pulmonary disease (TRIBUTE): a double-blind, parallel group, randomised controlled trial. Lancet.

[3] Bogart M, Stanford RH, Reinsch T, Hull M, Buikema A, Hulbert E (2018). Clinical characteristics and medication patterns in patients with COPD prior to initiation of triple therapy with ICS/LAMA/LABA: a retrospective study. Respir Med.

[4] Brusselle G, Price D, Gruffydd-Jones K, Miravitlles M, Keininger DL, Stewart R, Baldwin M, Jones RC (2015). The inevitable drift to triple therapy in COPD: an analysis of prescribing pathways in the UK. Int J Chron Obstruct Pulmon Dis.

[5] Vestbo J, Vogelmeier C, Small M, Higgins V (2014). Understanding the GOLD 2011 strategy as applied to a real-world COPD population. Respir Med.

[6] Watz H, Tetzlaff K, Wouters EFM, Kirsten A, Magnussen H, Rodriguez-Roisin R, Vogelmeier C, Fabbri LM, Chanez P, Dahl R (2016). Blood eosinophil count and exacerbations in severe chronic obstructive pulmonary disease after withdrawal of inhaled corticosteroids: a post-hoc analysis of the WISDOM trial. Lancet Respir Med.

[7] Chapman KR, Hurst JR, Frent SM, Larbig M, Fogel R, Guerin T, Banerji D, Patalano F, Goyal P, Pfister P (2018). Long-term triple therapy De-escalation to Indacaterol/Glycopyrronium in patients with chronic obstructive pulmonary disease (SUNSET): a randomized, double-blind, triple-dummy clinical trial. Am J Respir Crit Care Med.

[8] Clinical Practice Research Datalink. Accessed from: https://www.cprd.com/home. Accessed 1 Jan 2019.

[9] OptumInsight, Eden Prairie, MN, US. Available from: https://www.optum.com/content/dam/optum/resources/productSheets/Clinformatics-Data-Mart.pdf. Accessed 1 Jan 2019.

[10] Herrett E, Gallagher AM, Bhaskaran K, Forbes H, Mathur R, van Staa T, Smeeth L (2015). Data resource profile: clinical practice research datalink (CPRD). Int J Epidemiol.

[11] Global Strategy for the Diagnosis, Management and Prevention of COPD, Global Initiative for Chronic Obstructive Lung Disease (GOLD) 2017 Report. https://goldcopd.org/gold-2017-global-strategy-diagnosis-management-prevention-copd/. Accessed 1 Jan 2019.

[12] Mapel DW, Dutro MP, Marton JP, Woodruff K, Make B (2011). Identifying and characterizing COPD patients in US managed care. A retrospective, cross-sectional analysis of administrative claims data. BMC Health Serv Res.

[13] Macaulay D, Sun SX, Sorg RA, Yan SY, De G, Wu EQ, Simonelli PF (2013). Development and validation of a claims-based prediction model for COPD severity. Respir Med.

[14] Suissa S, Dell'Aniello S, Ernst P (2018). Comparative effectiveness of LABA-ICS versus LAMA as initial treatment in COPD targeted by blood eosinophils: a population-based cohort study. Lancet Respir Med.

[15] Rothnie KJ, Mullerova H, Smeeth L, Quint JK (2018). Natural history of chronic obstructive pulmonary disease exacerbations in a general practice-based population with chronic obstructive pulmonary disease. Am J Respir Crit Care Med.

[16] Calverley PMA, Tetzlaff K, Vogelmeier C, Fabbri LM, Magnussen H, Wouters EFM, Mezzanotte W, Disse B, Finnigan H, Asijee G (2017). Eosinophilia, frequent exacerbations, and steroid response in chronic obstructive pulmonary disease. Am J Respir Crit Care Med.

[17] Mapel D, Laliberte F, Roberts MH, Sama SR, Sundaresan D, Pilon D, Lefebvre P, Duh MS, Patel J (2017). A retrospective study to assess clinical characteristics and time to initiation of open-triple therapy among patients with chronic obstructive pulmonary disease, newly established on long-acting mono- or combination therapy. Int J Chron Obstruct Pulmon Dis.

[18] Oshagbemi OA, Burden AM, Braeken DCW, Henskens Y, Wouters EFM, Driessen JHM, Maitland-van der Zee AH, de Vries F, FME F (2017). Stability of blood eosinophils in patients with chronic obstructive pulmonary disease and in control subjects, and the impact of sex, age, smoking, and baseline counts. Am J Respir Crit Care Med.

[19] Shin SH, Park HY, Kang D, Cho J, Kwon SO, Park JH, Lee JS, Oh Y-M, Sin DD, Kim WJ (2018). Serial blood eosinophils and clinical outcome in patients with chronic obstructive pulmonary disease. Respir Res.

[20] Singh D, Kolsum U, Brightling CE, Locantore N, Agusti A, Tal-Singer R (2014). Eosinophilic inflammation in COPD: prevalence and clinical characteristics. Eur Respir J.

[21] Vestbo J, Anderson W, Coxson HO, Crim C, Dawber F, Edwards L, Hagan G, Knobil K, Lomas DA, MacNee W (2008). Evaluation of COPD longitudinally to identify predictive surrogate end-points (ECLIPSE). Eur Respir J.

[22] Bafadhel M, McKenna S, Terry S, Mistry V, Pancholi M, Venge P, Lomas DA, Barer MR, Johnston SL, Pavord ID (2012). Blood eosinophils to direct corticosteroid treatment of exacerbations of chronic obstructive pulmonary disease: a randomized placebo-controlled trial. Am J Respir Crit Care Med.

[23] Pascoe S, Locantore N, Dransfield MT, Barnes NC, Pavord ID (2015). Blood eosinophil counts, exacerbations, and response to the addition of inhaled fluticasone furoate to vilanterol in patients with chronic obstructive pulmonary disease: a secondary analysis of data from two parallel randomised controlled trials. Lancet Respir Med.

[24] Yun JH, Lamb A, Chase R, Singh D, Parker MM, Saferali A, Vestbo J, Tal-Singer R, Castaldi PJ, Silverman EK (2018). Blood eosinophil count thresholds and exacerbations in patients with chronic obstructive pulmonary disease. J Allergy Clin Immunol.

[25] Regan Elizabeth A., Hokanson John E., Murphy James R., Make Barry, Lynch David A., Beaty Terri H., Curran-Everett Douglas, Silverman Edwin K., Crapo James D. (2010). Genetic Epidemiology of COPD (COPDGene) Study Design. COPD: Journal of Chronic Obstructive Pulmonary Disease.

[26] Vedel-Krogh S, Nielsen SF, Lange P, Vestbo J, Nordestgaard BG (2016). Blood eosinophils and exacerbations in chronic obstructive pulmonary disease. The Copenhagen general population study. Am J Respir Crit Care Med.

[27] Global Strategy for the Diagnosis, Management and Prevention of COPD, Global Initiative for Chronic Obstructive Lung Disease (GOLD) 2019 Report. https://goldcopd.org/wp-content/uploads/2018/11/GOLD-2019-POCKET-GUIDE-FINAL_WMS.pdf. Accessed 1 Jan 2019.

[28] Kreindler JL, Watkins ML, Lettis S, Tal-Singer R, Locantore N (2016). Effect of inhaled corticosteroids on blood eosinophil count in steroid-Naïve patients with COPD. BMJ Open Respir Res.

[29] Roche N, Chapman KR, Vogelmeier CF, Herth FJF, Thach C, Fogel R, Olsson P, Patalano F, Banerji D, Wedzicha JA (2017). Blood eosinophils and response to maintenance chronic obstructive pulmonary disease treatment. Data from the FLAME trial. Am J Respir Crit Care Med.

