# Supplementary material for: Evaluation of exacerbations and blood eosinophils in UK and US COPD populations
Source: Respir Res. 2019 Aug 7;20:178. doi: 10.1186/s12931-019-1130-y (PMC6686508; doi:10.1186/s12931-019-1130-y)
Supplement: Supplementary file 1 — Figure S1. Study design. Table S1. Description of exacerbation algorithm. Table S2. Baseline demographics and clinical characteristics of 2 year follow-up subgroup of 2014 cohort. Table S3. Distribution of patients based on eosinophil counts (2014 cohort). Table S4. Patient flow (2015 cohort). Table S5. Baseline demographics and clinical characteristics. (2015 cohort). Table S6. COPD patient population by exacerbation frequency in the index year (2015 cohort). Table S7. Distribution of blood eosinophil count (2015 cohort). Table S8. Distribution of patients based on high exacerbations and eosinophil counts (2015 cohort). Table S9. Cross tabulation of frequency of eosinophil counts in 1st year and exacerbations in second year (2014 cohort). (DOCX 86 kb) [file 12931_2019_1130_MOESM1_ESM.docx]

**Supplementary Contents**

[**Additional file 1: Figure S1. Study design** 2](#_Toc13832244)

[**Additional file 1: Table S1. Description of exacerbation algorithm** 2](#_Toc13832245)

[**Additional file 1: Table S2. Baseline demographics and clinical characteristics of 2 year follow-up subgroup of 2014 cohort** 3](#_Toc13832246)

[**Additional file 1: Table S3. Distribution of patients based on eosinophil counts (2014 cohort)** 4](#_Toc13832247)

[**Additional file 1: Table S4. Patient flow (2015 cohort)** 5](#_Toc13832248)

[**Additional file 1: Table S5. Baseline demographics and clinical characteristics. (2015 cohort).** 5](#_Toc13832249)

[**Additional file 1: Table S6. COPD patient population by exacerbation frequency in the index year (2015 cohort)** 6](#_Toc13832250)

[**Additional file 1: Table S7. Distribution of blood eosinophil count (2015 cohort)** 7](#_Toc13832251)

[**Additional file 1: Table S8. Distribution of patients based on high exacerbations and eosinophil counts (2015 cohort)** 7](#_Toc13832252)

[**References** 9](#_Toc13832253)

# **Additional file 1: Figure S1. Study design**

| 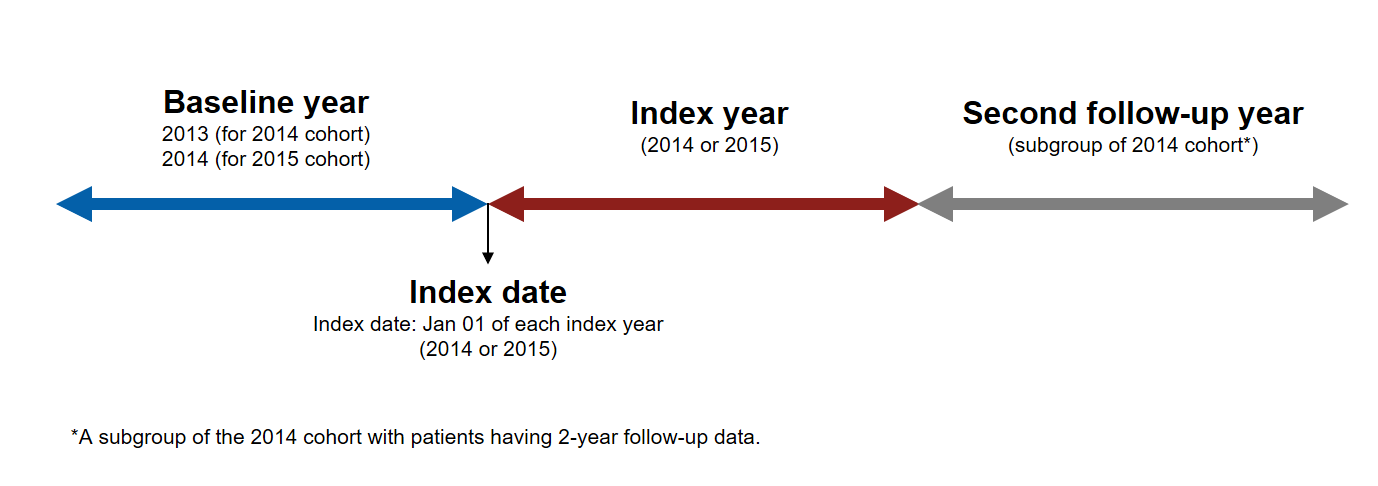 |
| --- |

# **Additional file 1: Table S1. Description of exacerbation algorithm**

| **Database** | **Algorithm** |
| --- | --- |
| **CPRD-COPD Exacerbation*** | - Episodes of severe exacerbation:   - A COPD-related hospital admission recorded in the HES with one of the following ICD-10 codes: J44.0, J44.1 in any diagnosis, or J44.9 in primary diagnosis, OR   - A COPD Emergency Department attendance recorded in the HES as diagnosis code 252 “Respiratory conditions – other non-asthma”, OR - Episodes of moderate exacerbation:   - A prescription for an OCS and an antibiotic for 5–14 days, prescribed on the same day, OR   - An exacerbation symptom or COPD diagnosis with a prescription for an OCS on the same day, OR   - An exacerbation symptom or COPD diagnosis with a prescription for an oral antibiotic on the same day, OR   - A lower respiratory tract infection code (excluding codes for pneumonia)   - Exacerbation symptoms are defined as codes suggesting an increase in two or more of the following: breathlessness, cough, sputum volume and purulence   - We also looked at an acute exacerbation recorded by a GP in the CPRD (Read codes: H312200, H3y1.00, 8BP8.00, 8H2R.00, 66Ye.00, 66Yd.00, 66Yi.00). |
| **OPTUM-COPD Exacerbation**** | - Hospital admission or emergency room visit with a primary diagnosis for COPD, OR - Outpatient visit with a diagnosis for COPD at any position with an OCS prescription order (one prescription for <30 days, no subsequent OCS prescription) within 7 days, OR - Outpatient visits associated with one of the following ICD-9 and ICD-10 codes in the first position:   - ICD-9 codes: 136.3, 466.19, 480-486, 487.0, 490, 491.21, 491.22, 494.1, 506.0-506.3, 507, 511.0-511.1, 512, 517.1, 518.0, 518.81, 518.82, and 518.84; OR   - ICD-10 codes: B59, J21.8, J21.9, J12, J13, J15, J16, J17, J18, J10, J11, J40, J44.1, J47.1, J68, J69, J94, R09.1, J93, J98.1, J96.0;OR - Outpatient visit with a diagnosis for COPD at any position with the following oral antibiotics claim (one prescription for < 30 days, no subsequent claim) within 7 days:   - amoxicillin, beta-lactamase inhibitors, second- or third-generation cephalosporins, macrolides, or doxycycline, respiratory fluoroquinolones or trimethoprim-sulfamethoxasole |

**Exacerbations occurring within 14 days were considered as a single event. Exacerbation in COPD patients was defined using a validated algorithm (Rothnie et al., 2016)^1^. As the algorithm only considers exacerbations treated in primary care (using the CPRD database), we integrated it with data on exacerbations treated in-hospital using the HES. Severe exacerbations were those associated with hospital admission or emergency room visit, and the others were classified under moderate exacerbations.*

***Events occurring within 14 days were considered the same exacerbation. Exacerbation in COPD patients was defined using a modified version of the algorithm developed by Mapel et al. and Macaulay et al.^,2,3^*

# **Additional file 1: Table S2. Baseline demographics and clinical characteristics of 2 year follow-up subgroup of 2014 cohort**

| **Characteristic** | **CPRD database, N=7,979** | **Optum database N=105,657** |
| --- | --- | --- |
| **Age, years, mean (SD)** | 71.1 (10.0) | 71.6 (10.5) |
| **Gender** | | |
| Men | 4,372 (54.8%) | 54,377 (51.5%) |
| Women | 3,607 (45.2%) | 51,280 (48.5%) |
| **Charlson Comorbidity Index*, median (IQR)** | 2.3 (1.0–3.0) | 2.2 (0.0–3.0) |
| **Baseline comorbidities common to COPD patients^†^** | | |
| Hypertension | 4,605 (57.7%) | 80,227 (75.9%) |
| Hyperlipidaemia | 3,042 (38.1%) | 69,973 (66.2%) |
| Depression | 2,715 (34.0%) | 18,669 (17.7%) |
| Anxiety | 2,307 (28.9%) | 16,784 (15.9%) |
| Cancer | 1,937 (24.3%) | 24,246 (23.0%) |
| Diabetes | 1,326 (16.6%) | 34,480 (32.6%) |
| **Baseline medications^‡^** | | |
| ICS | 618 (7.8%) | 4,308 (4.1%) |
| LABA | 247 (3.1%) | 1,112 (1.1%) |
| LAMA | 2021 (25.3%) | 18,342 (17.4%) |
| LAMA + LABA | 232 (2.9%) | 448 (0.4%) |
| ICS + LABA | 2,697 (33.8%) | 22,171 (21.0%) |
| ICS + LAMA + LABA | 3,044 (38.1%) | 7,637 (7.2%) |
| **Smoking status** | | |
| Patients with smoking data reported, n | 7,975 | — |
| Current smokers | 2,585 (32.4%) | — |
| Ex-smokers | 5,003 (62.7%) | — |
| Non-smokers | 387 (4.9%) | — |
| **GOLD classification^§^** | | |
| Patients with GOLD assessment, n | 3,265 | — |
| Group A | 219 (7.0%) | — |
| Group B | 980 (30.0%) | — |
| Group C | 169 (5.0%) | — |
| Group D | 1,897 (58.0%) | — |
| **mMRC dyspnoea scale** | | |
| Patients with mMRC data, n | 5,940 | — |
| Grade 0 | 739 (12.4%) | — |
| Grade 1 | 2,160 (36.4%) | — |
| Grade 2 | 1,752 (29.5%) | — |
| Grade 3 | 1,093 (18.4%) | — |
| Grade 4 | 196 (3.3%) | — |
| **Patients with FEV_1_ % predicted data** | 4,042 (50.7%) | — |
| FEV_1_, % predicted, mean (SD) | 61.8 (22.0) | — |
| **Patients with FEV_1_/FVC data** | 3,592 (45.0%) | — |
| FEV_1_/FVC, %, mean (SD) | 59.8 (16.6) | — |

# **Additional file 1: Table S3. Distribution of patients based on eosinophil counts (2014 cohort)**

| **Database** | **Eosinophil count in 2014** | **Eosinophil count in 2015 (second follow-up), n (%)** | |
| --- | --- | --- | --- |
|  |  | **<300 cells/μL** | **≥300 cells/μL** |
| **CPRD** | <300 cells/μL (N=2,379) | 1,990 (83.6%) | 389 (16.4%) |
|  | ≥300 cells/μL (N=1,093) | 395 (36.1%) | 698 (63.9%) |
| **Optum CDM** | <300 cells/μL (N=12,024) | 10,282 (85.50%) | 1,742 (14.50%) |
|  | ≥300 cells/μL (N=5,016) | 1,804 (36.00%) | 3,212 (64.00%) |

# **Additional file 1: Table S4. Patient flow (2015 cohort)**

|  | **CPRD database, N (%)** | **Optum CDM database, N (%)** |
| --- | --- | --- |
| **Total patients with existing COPD** | 26,445 (100%) | 378,969 (100%) |
| *Inclusion Reason:* |  |  |
| At least 40 years old on index date (Jan 01, 2014 or Jan 01, 2015) | 26,281 | 353,987 |
| Has continuous medical and pharmacy benefits 1 year pre-index date | 25,604 | 286,446 |
| Has continuous medical and pharmacy benefits 1 year post-index date | 15,477 | 219,009 |
| *Exclusion Criteria:* |  |  |
| Has diagnosis of asthma in either baseline or index year | 11,016 (35.0%) | 147,995 (39.1%) |
|  |  |  |
| **Total patients available for analysis*** |  |  |
| With 1-year follow-up | **11,016** | **147,995** |

**Patients with at least one COPD diagnosis during the index year period and met the eligibility criteria. CDM, Clinformatics™ Data Mart; CPRD, Clinical Practice Research Datalink*

# **Additional file 1: Table S5. Baseline demographics and clinical characteristics. (2015 cohort).**

| **Characteristic** | **CPRD database**  **N=11,016** | **Optum CDM database N=147,995** |
| --- | --- | --- |
| **Age, years, mean (SD)** | 71.8 (10.5) | 72.1 (10.6) |
| **Gender** | | |
| Men | 5,972 (54.2%) | 76,388 (51.6%) |
| Women | 5,044 (45.8%) | 71,607 (48.4%) |
| **Charlson Comorbidity Index*, Mean, SD** | 2.5 (2.2) | 2.00 (0.0–3.0) |
| **Baseline medications^‡^** | | |
| ICS | 5,851 (4.0%) | 5,900 (4.2%) |
| LABA | 1,434 (1.0%) | 1,556 (1.1%) |
| LAMA | 25,938 (17.5%) | 24,850 (17.8%) |
| LAMA + LABA | 789 (0.5%) | 615 (0.4%) |
| ICS + LABA | 32,136 (21.7%) | 29,763 (21.3%) |
| ICS + LAMA + LABA | 11,322 (7.7%) | 10,490 (7.5%) |
| **Smoking status** | | |
| Patients with smoking data reported, n | 11,011 | — |
| Current smokers | 659 (6.0%) | — |
| Ex-smokers | 6,805 (61.8%) | — |
| Non-smokers | 3,547 (32.2%) | — |
| **GOLD classification^§^** | | |
| Patients with GOLD assessment, n | 4,000 | — |
| Group A | 292 (7.3%) | — |
| Group B | 1,198 (30.0%) | — |
| Group C | 202 (5.1%) | — |
| Group D | 2,308 (57.7%) | — |
| **mMRC dyspnoea scale** | | |
| Patients with mMRC data, n | 7,573 | — |
| Grade 0 | 1,023 (13.5%) | — |
| Grade 1 | 2,697 (35.6%) | — |
| Grade 2 | 2,237 (29.5%) | — |
| Grade 3 | 1,357 (17.9%) | — |
| Grade 4 | 259 (3.4%) | — |
| **Patients with FEV1 % predicted data** | **4,958 (45.0%)** | — |
| FEV1, % predicted, mean (SD) | 60.3 (16.7) | — |
| **Patients with FEV1/FVC data** | **4,707 (42.7%)** | — |
| FEV1/FVC, %, mean (SD) | 60.2 (16.0) | — |

*Data are presented as n (%), unless specified otherwise. *Charlson Comorbidity Index comprises 19 comorbid disease categories, each assigned a score from 1–6, and is used to predict 10-year mortality in patients with comorbidities; the greater the score, the greater the risk of mortality. ^‡^Patient was only counted if length of medication use was ≥30 days. ^§^As per GOLD 2017 recommendations^4^*

*CDM, Clinformatics™ Data Mart; COPD, chronic obstructive pulmonary disease; CPRD, Clinical Practice Research Datalink; FEV_1_, forced expiratory volume in 1 second; FVC, forced vital capacity; GOLD, Global Initiative for Chronic Obstructive Lung Disease; ICS, inhaled corticosteroid; IQR, interquartile range; LABA, long-acting β2-agonist; LAMA, long-acting muscarinic antagonist; mMRC, modified Medical Research Council; SD, standard deviation*

# **Additional file 1: Table S6. COPD patient population by exacerbation frequency in the index year (2015 cohort)**

| **Number of Index Year Exacerbation** | **CPRD database**  **N=11,016** | **Optum CDM database N=147,995** |
| --- | --- | --- |
| **0** | 5,431 (49.3%) | 53,462 (36.1%) |
| **1** | 2,325 (21.1%) | 38,118 (25.8%) |
| **≥2** | 3,260 (29.6%) | 56,415 (38.1%) |

# **Additional file 1: Table S7. Distribution of blood eosinophil count (2015 cohort)**

| **Baseline EOS Count* (N,%)** | **CPRD database**  **N=11,016** | **Optum CDM database N=147,995** |
| --- | --- | --- |
|  |  |  |
| Patient has EOS count | 6,706 (60.9%) | 40,264 (27.2%) |
| <150 | 2,350 (35.0%) | 15,013 (37.3%) |
| 150-299 | 2,205 (32.9%) | 14,540 (36.1%) |
| 300-399 | 1,044 (15.6%) | 5,489 (13.6%) |
| ≥400 | 1,107 (16.5%) | 5,222 (13.0%) |

**The closest eosinophil record within ±180 days to the index date was used as the baseline value*

# **Additional file 1: Table S8. Distribution of patients based on high exacerbations and eosinophil counts (2015 cohort)**

| **Population** | **CPRD database**  **N=11,016** | **Optum CDM database N=147,995** |
| --- | --- | --- |
| **Patients with ≥2 exacerbations in index year, n** | 3,260 | 56,415 |
| Patients with eosinophil count in baseline period, n | 2,106 | 15,346 |
| <150 cells/μL | 757 (35.9%) | 5,777 (37.6%) |
| 150-299 cells/μL | 666 (31.6%) | 5,464 (35.6%) |
| 300-399 cells/μL | 343 (16.3%) | 2,066 (13.5%) |
| ≥400 cells/μL | 340 (16.1%) | 2,039 (13.3%) |
| **Total patients with eosinophil count in baseline period, n** | 6,706 | 40,264 |
| ≥2 exacerbations eosinophil count ≥300 cells/μL, n (%) | 683 (10.2%) | 4,105 (10.2%) |
| ≥2 exacerbations eosinophil count ≥400 cells/μL, n (%) | 340 (5.1%) | 2,039 (5.1%) |

**Additional file 1: Table S9. Cross tabulation of frequency of eosinophil counts in 1st year and exacerbations in second year (2014 cohort).**

| **2014 eosinophil count** | **2015 exacerbation** | | | | |
| --- | --- | --- | --- | --- | --- |
|  | **0** | **1** | **2** | **3+** | **Total** |
| **UK CPRD database, n (%)** | | | | | |
| <150 | 527 (42.19%) | 285 (22.82%) | 176 (14.09%) | 261 (20.90%) | 1,249 (100%) |
| 150-299 | 474 (41.95%) | 268 (23.72%) | 147 (13.01%) | 241 (21.33%) | 1,130 (100%) |
| 300+ | 420 (38.43%) | 286 (26.17%) | 137 (12.53%) | 250 (22.87%) | 1,093 (100%) |
| **US Optum database, n (%)** | | | | | |
| <150 | 2,500 (37.29%) | 1,739 (25.94%) | 1,074 (16.02%) | 1,392 (20.76%) | 6,705 (100%) |
| 150-299 | 1,954 (36.74%) | 1,313 (24.69%) | 913 (17.16%) | 1,139 (21.41%) | 5,319 (100%) |
| 300+ | 1,710 (34.09%) | 1,398 (27.87%) | 773 (15.41%) | 1,135 (22.63%) | 5,016 (100%) |
| Total | 6,164 | 4,450 | 2,760 | 3,666 | 17,040 |

# **References**

1. Rothnie KJ, Müllerová H, Hurst JR, *et al.* Validation of the Recording of Acute Exacerbations of COPD in UK Primary Care Electronic Healthcare Records. *PloS one* 2016;11:e0151357
2. Mapel DW, Dutro MP, Marton JP, *et al.* Identifying and characterizing COPD patients in US managed care. A retrospective, cross-sectional analysis of administrative claims data. *BMC Health Serv Res* 2011;11:43.
3. Macaulay D, Sun SX, Sorg RA, *et al.* Development and validation of a claims-based prediction model for COPD severity. *Respir Med* 2013; 107(10):1568-1577*.*
4. Global Strategy for the Diagnosis, Management and Prevention of COPD, Global Initiative for Chronic Obstructive Lung Disease (GOLD) 2017 Report. <https://goldcopd.org/wp-content/uploads/2016/12/wms-GOLD-2017-Pocket-Guide.pdf> (accessed 1 Jan 2019).
